# Transcriptional Response of *Burkholderia cenocepacia* H111 to Severe Zinc Starvation

**DOI:** 10.3389/bjbs.2023.11597

**Published:** 2023-09-26

**Authors:** James Paul Barnett

**Affiliations:** College of Life Sciences, Birmingham City University, Birmingham, United Kingdom

**Keywords:** *Burkholderia*, RNA-seq, zinc homeostasis, nutritional immunity, Zur

## Abstract

*Burkholderia cenocepacia* is an opportunistic pathogen that is primarily associated with severe respiratory infections in people with cystic fibrosis. These bacteria have significant intrinsic resistance to antimicrobial therapy, and there is a need for more effective treatments. Bacterial zinc uptake and homeostasis systems are attractive targets for new drugs, yet our understanding of how bacteria acquire and utilise zinc remains incomplete. Here we have used RNA-sequencing and differential gene expression analysis to investigate how *B. cenocepacia* H111 is able to survive in zinc poor environments, such as those expected to be encountered within the host. The data shows that 201 genes are significantly differentially expressed when zinc supply is severely limited. Included in the 85 upregulated genes, are genes encoding a putative ZnuABC high affinity zinc importer, two TonB-dependent outer membrane receptors that may facilitate zinc uptake across the outer cell membrane, and a COG0523 family zinc metallochaperone. Amongst the 116 downregulated genes, are several zinc-dependent enzymes suggesting a mechanism of zinc sparring to reduce the cells demand for zinc when bioavailability is low.

## Introduction

The *Burkholderia cepacia complex* (*Bcc*) is a group of at least 24 species of bacteria widely distributed in the environment [1]. They first came to prominence as opportunistic pathogens in the 1980s, causing severe respiratory infections in people with cystic fibrosis (CF) [2]. Whilst all species within the *Bcc* have been identified in sputum from CF patients, *B. cenocepacia* and *B. multivorans* are responsible for most infections, accounting for 85%–97% of cases [3]. *Bcc* infections in CF patients have a variable clinical course, although the prognosis is poor. Infections often result in an accelerated decline in pulmonary function and increased morbidity and mortality. *Bcc* bacteria can also lead to the development of “cepacia syndrome” that is characterised by a fatal necrotising pneumonia and bacteraemia [4]. *Bcc* bacteria are naturally resistant to major classes of antibiotics in clinical use, making infections particularly difficult to eradicate [5]. There are currently no standardised drug regimens/guidelines for the treatment of these infections and no vaccines available [6]. The introduction of strict infection control measures and the segregation of patients has resulted in a marked reduction in patient-to-patient transmissions in recent decades [7]. The epidemiology of *Bcc* bacteria is not limited to CF, with infections also occurring in people with chronic granulomatous disease [8], and regular reports of nosocomial infections in both immune compromised and immune competent individuals, often from contaminated medical supplies [9].

To survive, pathogens must be able to acquire the essential nutrients they need for growth in competition with the host. Zinc is an essential nutrient for bacteria, functioning as a catalytic cofactor or structural component of numerous proteins [10]. It is involved in fundamental cellular processes including DNA replication, regulation of gene expression and protection against oxidative damage. In some bacteria, zinc also plays a direct role in antibiotic resistance, with the production of zinc-dependent β-lactamases [11]. Overall, 5%–6% of the proteins encoded in any bacterial genome are thought to bind zinc [10]. However, since free zinc can also be toxic when present in excess, cellular zinc levels are carefully controlled by a combination of import, efflux, and storage [12]. During infection, the host attempts to deprive bacteria of the essential zinc they need to grow in a process called nutritional immunity [13]. A key aim of this study was to determine how *B. cenocepacia* can acquire zinc and survive in environments where zinc supply is severely restricted.

Almost all bacteria studied thus far possess ATP-binding cassette (ABC) transporters (ZnuABC) for zinc uptake in zinc poor environments [14]. These tripartite systems consist of a Zn^2+^ binding periplasmic protein (ZnuA), a membrane spanning permease (ZnuB) that facilitates Zn^2+^ transport across the plasma membrane, and a cytosolic ATPase (ZnuC) that drives transport [14]. Expression of *znuABC* is dependent upon intracellular zinc status and is regulated by the transcriptional repressor protein Zur [14]. In its fully Zn^2+^ bound state, Zur binds to specific DNA sequences (Zur boxes), within the operator regions of regulated genes. This prevents the transcription of downstream genes including *znuABC* avoiding the accumulation of toxic levels of zinc inside the cell [12, 14]. Some bacterial species have an additional Zur regulated protein called ZinT that acts in concert with ZnuA in the recruitment of Zn^2+^ within the periplasm [15].

A few bacterial species have been discovered to have additional transporters for zinc uptake across the plasma membrane. *Haemophilus influenzae* encodes at least two high affinity zinc uptake systems, (ZnuABC and a novel ZevAB system) [16], whilst *Escherichia coli* and *Salmonella enterica* both have a constitutively expressed low affinity ZIP-family transporter (ZupT) that import zinc under metal replete conditions [17].

It is largely assumed that passive diffusion of zinc through unspecific channels in the outer cell membrane of Gram-negative bacteria occurs in replete environments. However, this is unlikely to meet cellular demand when zinc supply is severely restricted. One potential strategy that bacteria might use to compete with the host for zinc is to secrete small molecules called “zincophores” that are like siderophores used for iron acquisition. To date, very few bacterial “zincophores” have been identified. A broad-spectrum nicotinamine-like metallophore (staphylopine) is produced by *Staphylococcus aureus* and is involved in the uptake of several metals, including zinc [18, 19]. In *Yersinia pestis*, there is evidence for the involvement of the siderophore yersiniabactin in zinc uptake [20] and in *Pseudomonas aeruginosa* a *bona fide* “zincophore” (pseudopaline) has been discovered that is regulated by Zur [21]. Several Gram-negative bacteria have TonB-dependent outer membrane receptors (e.g., ZnuD in *Neisseria meningitidis*), that are regulated by Zur and involved in zinc uptake [22]. It remains unclear in what form zinc is transported via these systems, but it is tempting to postulate that they may deliver “zincophores” into the cell. More recently, a type VI secretion system in both *Burkholderia thailandenis* and *Yersinia pseudotuberculosis* was found to play a role in scavenging zinc specifically during oxidative stress [23, 24].

Here we report the effect of zinc-depletion on growth, cellular zinc quotas, and gene transcription in *B. cenocepacia* H111.

## Materials and Methods

### Bacterial Strains and Growth Conditions

*Burkholderia cenocepacia* H111 was obtained from the Belgian co-ordinated collection of microorganisms. Cultures were grown in either LB-broth (Millers), or a modified Glycerol-glycerophosphate medium (GMM) with or without the addition of zinc [25]. Cultures were routinely grown aerobically at 37°C within an orbital shaking incubator.

The composition of GGM medium was as follows: 40 mM MES, 18.7 mM NH_4_Cl, 13.4 mM KCl, 7.64 mM β-glycerophosphate, 5.0 mM glycerol, 4.99 mM K_2_SO_4_, 1.0 mM MgCl_2_, 0.134 mM EDTA, 68.0 µM CaCl_2_·2H_2_0, 18.5 µM FeCl_3_·6H_2_O, 12.28 µM ZnSO_4_·7H_2_O, 1.62 µM H_3_BO_3_, 0.5 µM MnCl_2_·4H_2_O, 587 nM CuCl_2_·2H_2_O, 344 nM Co(NO_3_)_2_·6H_2_O, and 80.9 nM (NH_4_)6Mo_7_O_24_·4H_2_O, in MilliQ (18 MΩ cm^−1^) water. To prepare the medium, bulk components (MES, NH_4_Cl, KCl, K_2_SO_4_, glycerol, H_3_BO_3_), dissolved in MilliQ water were passed through a Chelex 100 column (Bio Rad, United Kingdom) to remove contaminating metal cations, following the manufacturer’s instructions. CaCl_2_ was subsequently added, and the solution sterilised in polycarbonate bottles using a microwave, to avoid introduction of contaminating zinc [26]. After sterilisation, trace metals dissolved in EDTA, MgCl_2_, and β-glycerophosphate were added by filter sterilisation using 0.2 µm PTFE syringe filters. Syringes without rubber seals were used to minimise zinc contamination. Chemicals were purchased from either Merck or Fisher Scientific and were trace metal grade where available. To further minimise the potential for zinc contamination, culture flasks, measuring cylinders, and other plasticware was washed in 3% trace metal grade nitric acid overnight, and extensively rinsed using MilliQ water (18 MΩ cm^−1^) before use.

### Growth Curves

Ten mL of overnight cultures of *B. cenocepacia* H111 in LB, were harvested by centrifugation at 3,000 *g* for 15 min, and the cell pellets washed by gently resuspending in GGM without trace metal additions but supplemented with 1 mM EDTA to remove surface adsorbed metals. Cells were pelleted again and further washed in trace metal free GGM to remove EDTA. After a final centrifugation step, cells were resuspended in 10 ml of complete GGM with or without the addition of zinc. Twenty µL of the resuspended culture was added to 180 µl of fresh GGM in 96-well plates. Plates were sealed using Breathe-Easy^®^ plate seals and incubated at 37°C with orbital shaking at 200 rpm in a CLARIOstar plate reader (BMG LABTECH). Optical density (600 nm) measurements were taken every 10 min for 20 h.

### Inductively Coupled Plasma Mass Spectrometry (ICP-MS)

*B. cenocepacia* H111 was cultured in zinc depleted and zinc replete GGM and harvested at late exponential growth phase, by centrifugation at 3,000 *g*. Cell pellets were washed with EDTA as described above to remove any surface adsorbed metals. After washing, the cells were collected by centrifugation and dried to constant weight before being digested in 70% trace metal grade nitric acid for 7 days at room temperature. Following complete digestion, samples were diluted to 2% nitric acid in MilliQ water and passed through 0.45 µm PTFE filters. ICP-MS analysis was performed using a NexION 300X instrument (PerkinElmer). Calibration was performed with an external ^66^Zn standard across the 0–1,000 ppb range. ^45^Sc was used as the internal standard. The instrument was operated in Helium KED mode to remove any polyatomic interferences. Five technical replicates were taken for each sample analysed, and four biological replicates were performed.

### RNA-Sequencing

RNA-sequencing and differential gene expression analysis was performed by GENEWIZ™. *B. cenocepacia* H111 was grown in zinc depleted and zinc replete GGM to mid-exponential growth phase, as described above. Cells were harvested by centrifugation at 3,000 *g*, and cell pellets stored at −80°C for 1 week before shipping on dry-ice to GENEWIZ™ for RNA extraction, RNA library preparation and RNA-sequencing. Total RNA was extracted using a Qiagen RNeasy Plus Mini Kit. rRNA depletion was performed using Qiagen FastSelect rRNA bacteria kit. RNA quantity and integrity were determined using a Qubit 2.0 Fluorometer, and Agilent 4200 Tapestation. RNA sequencing libraries of 150 bp reads, were prepared using a NEBNext Ultra II RNA library kit. Samples were paired end sequenced using an Illumina Hiseq instrument.

### Data Processing and Differential Gene Expression Analysis

Data processing was performed by GENEWIZ™. RNA-sequence reads were trimmed using Trimmomatic v. 0.36, removing adapter sequences. The trimmed reads were mapped to the *B. cenocepacia* H111 reference genome [27], using STAR aligner v. 2.5.2b. Unique gene hit counts were calculated by using featureCounts v.1.5.2. Only unique reads within exon regions were counted. Gene hit counts were used for downstream differential expression analysis. Using DESeq2, a comparison of gene expression between cells grown in zinc replete and zinc depleted GGM was performed. *p*-values and Log2 fold changes were calculated using the Wald test. Genes with adjusted *p*-values < 0.05 and absolute log2 fold changes > 1 were called as being differentially expressed.

### RT-qPCR

RNA was extracted from cell pellets using a GeneJET RNA Purification kit (Thermo Scientific™) and used a template for RT-qPCR. Gene sequences were extracted from the *Burkholderia* genome database [27], and primers designed using Primer3 v. 4.1.0 [28]. A full list of primers used is given in Supplementary Table S1. RT-qPCR was performed using Power SYBR™ green RNA-to-C_T_ 1-step kit (Applied Biosytems™), following the manufacturer’s instructions. PCR was performed using a Quantstudio 3 real time PCR system (Applied Biosystems™). Cycling was as follows: 1 cycle of 48°C for 30 min, 1 cycle of 95°C for 10 min, 40 cycles of 95°C for 15 s and 60°C for 60 s. Relative levels of gene expression were calculated using the 2^−ΔΔCt^ method using the *gyrB* gene as an internal control.

## Results

### Effect of Zinc Depletion on Growth of *B. cenocepacia* H111

**FIGURE 1:**
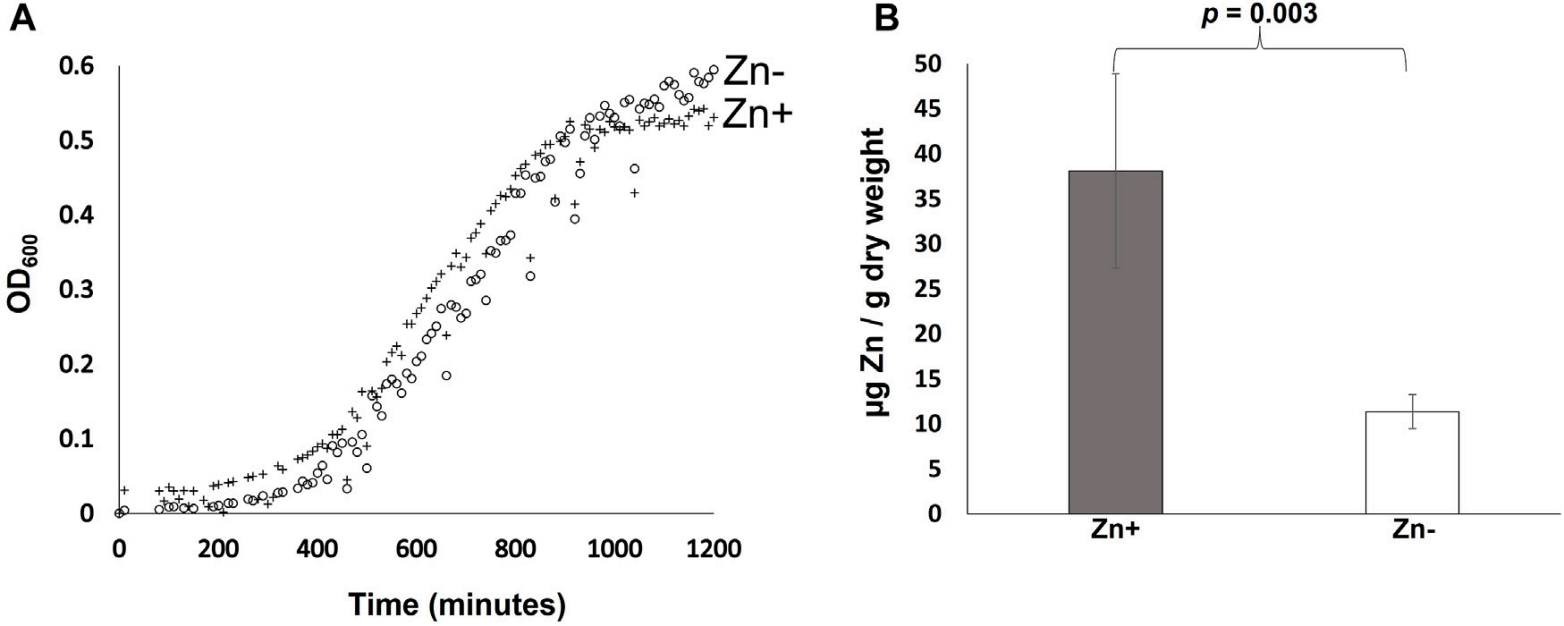
Effect of zinc availability on growth and cellular zinc quotas of *B. cenocepacia* H111. **(A)** Growth of *B. cenocepacia* H111 in zinc-replete GGM (crosses), and zinc-depleted GGM (open circles), over 20 h. OD_600_ values measured as a proxy for cell numbers. Mean values from 12 replicate samples are plotted. **(B)**. Total cellular zinc quotas (expressed as µg zinc per g of dry cell weight) in *B. cenocepacia* H111 following culture in zinc-replete GGM (grey bar), and zinc-depleted GGM (white bar). Mean values from 4 independent biological replicates are plotted, with error bars showing standard deviations. The *p*-value from a two-tailed *t*-test is given.

*B. cenocepacia* H111 was cultured in zinc replete and depleted GGM and growth monitored using OD_600_ measurements as a proxy for cell numbers. The data (Figure 1A) reveals no significant difference in growth between the two conditions evaluated. The duration of the lag phase is comparable, and cells grown in zinc depleted and replete media reached similar final cell numbers with an average OD_600_ of 0.54 and 0.59, respectively at 20 h. Average doubling times during exponential growth across both conditions was 226 min. That compares to a doubling time in LB-broth of 108 min (data not shown). Growth rates in LB in our lab are comparable to previously reported doubling times for *Bcc* species of between 70–186 min [29]. The result of this experiment is significant for two reasons. Firstly, the data shows that *B. cenocepacia* H111 is well adapted for growth in zinc-depleted environments such as those expected to be encountered in the host, and secondly because it demonstrates that GGM-medium is suitable for trace metal studies in *Burkholderia* species more generally.

### Effect of Zinc Depletion on Total Cellular Zinc Quotas

The effect of zinc availability on total cellular zinc quotas was determined using Inductively coupled plasma mass spectrometry (ICP-MS). The data shows that that cellular zinc quotas are significantly reduced when *B. cenocepacia* H111 cells are grown in zinc depleted GGM compared to when they are grown in the replete medium (Figure 1B). Cells grown in the zinc replete GGM contained an average of 38.10 µg of zinc per g of dry cell weight, compared to just 11.35 µg of zinc per g of dry cell weight in cells grown in the zinc depleted medium. This result is surprising when taken in the context of the previous growth experiment that showed zinc depletion had no measurable effect on growth rates (Figure 1A), and together, these data suggest that *B. cenocepacia* H111 may either maintain significant zinc stores within the cell during replete conditions, or use “zinc sparing” as a mechanism to reduce overall cellular demand for zinc, when bioavailability is low. This phenomenon has been observed in other bacterial species where the expression of genes encoding non-essential zinc dependent proteins is repressed in response to zinc depletion [30].

### Changes in Gene Expression in Response to Zinc Depletion

**FIGURE 2:**
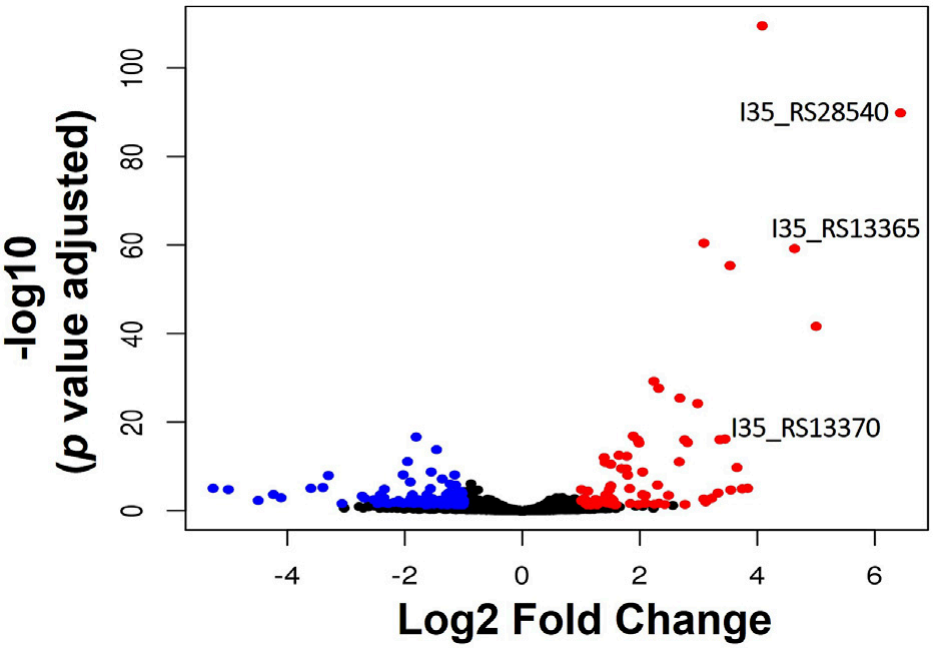
Transcriptional response of *B. cenocepacia* H111 to zinc depletion. Volcano plot showing the number of genes significantly upregulated (red circles), or downregulated (blue circles) in *B. cenocepacia* H111 in response to zinc depletion. Genes encoding a putative zinc metallochaperone (locus-tag I35_RS28540), of the COG0523 subfamily of P-loop GTPases, putative Zur-transcriptional regulator (locus-tag I35_RS13365), and putative ZnuA periplasmic Zn-binding protein (locus-tahgI35_RS13370) are indicated.

**FIGURE 3:**
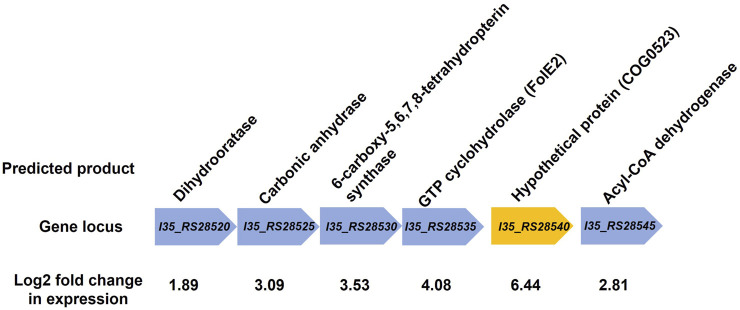
Genomic neighbourhood of putative *COG0523* gene. Gene encoding the putative COG0523 zinc metallochaperone is shown (orange arrow), along with neighbouring genes. Gene locus tag is given along with the predicted gene product from the *Burkholderia* genome database [27]. All genes shown are upregulated in response to zinc depletion, and the Log2 fold change in gene expression is shown.

RNA-sequencing was used to measure any changes in gene expression that occur in *B. cenocepacia* H111 in response to zinc depletion. A total of 201 genes showed significant differential expression, with 85 upregulated and 116 downregulated genes (Figure 2), representing 2.95% of predicted genes. The full list of significantly differentially expressed genes is given in Supplementary Table S2, and the 33 genes that had more than a Log2 fold increase in expression, are also listed in Table 1. The gene with the greatest fold increase in expression (locus tag *I35_RS28540*) encodes a hypothetical cytoplasmic protein [27]. A protein BLAST of the translated gene sequence reveals that the gene encodes a protein with significant sequence similarity to members of the COG0523 subfamily of P-loop GTPases that are predicted to play a role in metal homeostasis [31]. In the opportunistic pathogen *Acinetobacter baumannii*, the orthologous gene *zigA* is zinc binding and required for growth under zinc limited conditions [32]. ZigA from *A*. *baumannii* and the putative COG0523 protein from *B. cenocepacia* H111 share 75% sequence identity, and a conserved metal binding motif (CICC). Closer examination of the genomic neighbourhood of the *COG0523* gene in *B. cenocepacia*, reveals that it sits in a cluster of several genes that are upregulated in response to zinc depletion (Figure 3; Table 1; Supplementary Table S2), and includes genes that encode the usually zinc dependent proteins carbonic anhydrase, dihydrooratase, and 6-carboxy-5,6,7,8-tetrahydropterin synthase (QueD), as well as FolE2, and Acyl-coA dehydrogenase.This data points to a key role for *COG0523* in the response of *B. cenocepacia* to zinc limitation.

**TABLE 1:** List of genes with more than Log2-fold increase in expression in response to zinc depletion.

Gene locus	Gene product (from the *Burkholderia* genome database) [27]	Log2 fold change	*p*-value adjusted
I35_RS28540	Hypothetical protein	6.436363914	1.52E−90
I35_RS16050	Alpha/beta hydrolase	5.000537573	2.42E−42
I35_RS13365	Fur family transcriptional regulator	4.633320929	6.65E−60
I35_RS28535	GTP cyclohydrolase	4.083416486	3.42E−110
I35_RS01025	Phenylacetic acid degradation protein	3.833347834	0.00000913
I35_RS10880	Histidine ammonia-lyase	3.743017919	0.0000121
I35_RS01000	4-hydroxypehnylpyruvate dioxygenase	3.652890929	1.84E−10
I35_RS01030	Phenylacetate-CoA oxygenase subunit PaaJ	3.547319093	0.0000209
I35_RS28530	6-carboxy-5,6,7,8-tetrahydropterin synthase	3.534715892	4.48E−56
I35_RS13370	Cation ABC transporter substrate-binding protein	3.449536319	6.4E−17
I35_RS16055	Amino acid ABC transporter ATP-binding protein	3.362442596	8.91E−17
I35_RS10875	Histidine utilization repressor	3.330074112	0.0000992
I35_RS01035	Phenylacetic acid degradation protein	3221187039	0.001583599
I35_RS10870	Urocanate hydratase	3.121275534	0.010056873
I35_RS28525	Carbonate dehydratase	3.090073095	3.69E−61
I35_RS01040	Phenylacetate-CoA oxygenase subunit PaaB	3.088767817	0.002296074
I35_RS13375	ABC transporter ATP-binding protein	2.983146959	6.31E−25
I35_RS28545	Acyl-CoA dehydrogenase	2.809792771	4.11E−16
I35_RS13620	MFS transporter	2.768646664	0.039419489
I35_RS06170	TonB-dependent receptor	2.762808558	1.03E−16
I35_RS29380	Glyoxalase	2.680789605	3.92E−26
I35_RS23575	TonB-dependent receptor	2.48708683	0.000346391
I35_RS18920	Hypothetical protein	2.423450935	0.039764095
I35_RS10860	Imidazolonepropionase	2.326828435	0.022280782
I35_RS06165	Phenol degradation protein	2.320499563	2.27E−28
I35_RS22340	Hypothetical protein	2.300982216	0.00000167
I35_RS20825	Alpha/beta hydrolase	2.246852811	0.035832439
I35_RS13380	ABC transporter permease	2.238628283	5.97E−30
I35_RS10865	Hypothetical protein	2.099694274	0.022469745
I35_RS16595	DNA-binding protein	2.091617711	0.000362322
I35_RS29435	Hypothetical protein	2.072176035	0.022280782
I35_RS27035	2,2-Dialkylglycine decarboxylase	2.047152459	0.00024177
I35_RS16045	Hypothetical protein	2.046747087	1.75E−09

**FIGURE 4:**
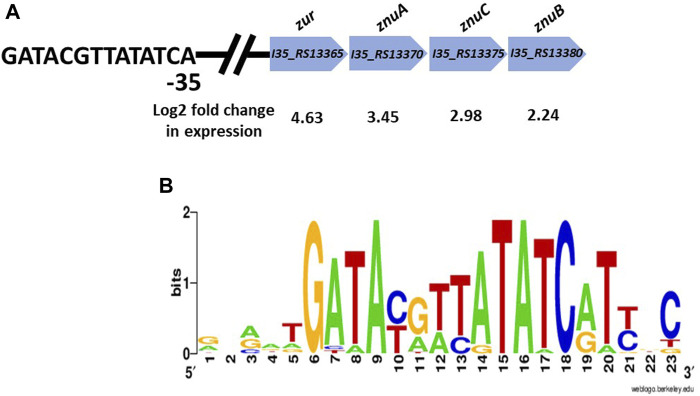
**(A)** The genomic arragement of the putative *znuACB* operon in *B. cenocepacia* H111 is presented, along with the predicted *zur* gene and Zur-binding site. Log2 fold change in gene expression in response to zinc depletion is shown. **(B)** Zur-box motif for *Burkholderia* species taken from the RegPrecise database [33].

The RNA-seq data also revealed a set of upregulated genes encoding a “cation ABC transporter,” and a “Fur-family transcriptional regulator”, which based on protein BLAST searches are likely to encode the high affinity zinc uptake transporter ZnuABC and the zinc transcriptional repressor protein Zur. Figure 4A shows the genomic arrangement of these genes, and a putative Zur binding site that was identified upstream of *zur* that matches exactly to a consensus Zur-box motif for *Burkholderia* taken from the RegPrecise database (Figure 4B) [33]. It should be noted that it can be difficult to accurately determine the metal specificity of proteins using bioinformatics, and this is the first experimental evidence that this is the ZnuABC system in *B. cenocepacia*.

Other upregulated genes identified that may play a role in zinc uptake from depleted environments include two TonB-dependent outer membrane receptors and a Major Facilitator Superfamily (MFS) transporter (Table 1), although a specific role for these transporters in zinc uptake remains to be experimentally confirmed.

Intriguingly, the expression of genes related to phenylacetic acid degradation were significantly upregulated too (Table 1), with this catabolic pathway required for the full pathogenicity of *B. cenocepacia* in a *Caenorhabditis elegans* infection model [34].

Amongst the genes that are downregulated in response to zinc depletion are some genes encoding usually zinc dependent proteins including aldolase, peptidase M23 and a dihydroorotase (Table 2; Supplementary Table S2), providing further evidence that *B. cenocepacia* H111 uses a mechanism of zinc sparing to reduce overall cellular demand for zinc during conditions of zinc deprivation. Also noteworthy was the downregulation of a membrane ion permease and a predicted metal transporting ATPase, possibly to reduce zinc efflux from the cell (Table 2; Supplementary Table S2).

**TABLE 2:** List of genes with more than Log2 fold reduction in expression in response to zinc depletion.

Gene locus	Gene product (from the *Burkholderia* genome database) [27]	Log2 fold change	*p*-value adjusted
I35_RS05820	Carbamoyl phosphate synthase small subunit	−2.024863193	0.023635014
I35_RS04670	D-amino acid dehydrogenase	−2.027861444	7.77E−09
I35_RS00160	F0F1 STP synthase subunit C	−2.0890612	0.007801604
I35_RS03110	Anthranilate synthase	−2.095343988	0.016002175
I35_RS34475	LysR-family transcriptional regulator	−2.107024951	0.005429617
I35_RS00155	ATP synthase FoF1 subunit A	−2.183650426	0.013504754
I35_RS07930	Peptidase M23	−2.283848351	0.027058355
I35_RS08110	Uroporphyrin-III C-methyltransferase	−2.348103878	0.0000143
I35_RS13745	MIP family channel protein	−2.34927356	0.015213303
I35_RS04075	Hypothetical protein	−2.355094182	0.00130666
I35_RS10765	Glutamine synthetase	−2.366860655	0.01636581
I35_RS00165	ATP synthase subunit B	−2.411303669	0.003024043
I35_RS14515	Dioxygenase	−2.415249229	0.000261085
I35_RS32500	Spermidine synthase	−2.433711956	0.001790561
I35_RS03095	Thioredoxin	−2.460179293	0.027058355
I35_RS12715	Uroporphyrin-III C-methyltransferase	−2.527603678	0.003439743
I35_RS16655	n-acetyl-gamma-glutamyl-phosphate reductase	−2.686444773	0.001583599
I35_RS22845	Lysine transporter LysE	−2.724901267	0.000529129
I35_RS10190	RNA helicase	−3.068648173	0.025920137
I35_RS32515	Hypothetical protein	−3.300579506	1.14E−08
I35_RS32510	Aldolase	−3.393813782	0.00000587
I35_RS32505	Alkylphosphonate utilization protein	−3.59778736	0.00000913
I35_RS17295	Hypothetical protein	−4.106261473	0.001250066
I35_RS13740	Glycerol kinase	−4.235597783	0.000223388
I35_RS23825	Translation initiation factor IF-1 2	−4.495477483	0.005043043
I35_RS25975	Diaminopimelate decarboxylase	−5.003385836	0.0000168
I35_RS13735	Glycerol-3-phosphate dehydrogenase	−5.263061988	0.00000913

**FIGURE 5:**
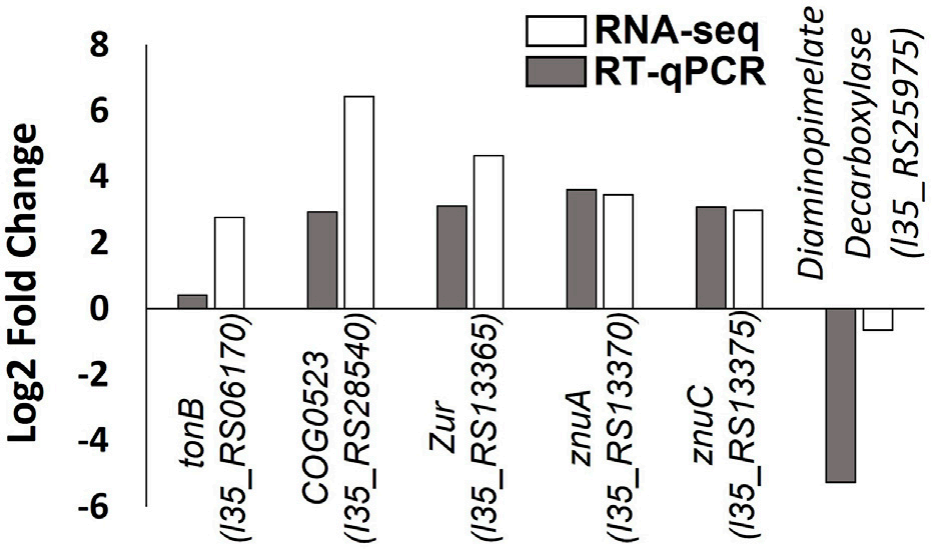
Validation of RNA-seq data by RT-qPCR. Log2 fold change in gene expression of six genes is plotted, with RNA-seq data shown with white bars and RT-qPCR data shown in grey bars. Gene locus tags and protein products are given.

The RNA-seq data were validated using RT-qPCR to independently measure changes in the expression of six genes in response to zinc depletion. Several of the genes selected (*tonB-dependent receptor*, *COG0523*, *zur*, *znuA* and *znuC*), are putative components of the zinc homeostasis system in *B. cenocepacia* H111 identified in this study. Good agreement was observed between the two data sets (Figure 5).

## Discussion

Species of bacteria belonging to the *B. cepacia complex* (*Bcc*), are most well-known for causing severe respiratory infections in patients with cystic fibrosis and are associated with poor patient outcomes [1–4]. These bacteria have significant intrinsic resistance to major classes of antibiotics, making infections particularly difficult to clear [5, 6]. Despite this and owing to the relatively small numbers of people affected by these bacteria, they have been historically understudied compared to many other human pathogens.

During an infection, our innate immune systems work to starve bacteria of key nutrients including iron and zinc to restrict their growth in the body [13]. Given that zinc and other metals are essential micronutrients for bacteria, metal uptake and homeostasis systems are attractive targets for novel antimicrobial pharmaceuticals. However, a detailed understanding of bacterial metal homeostasis is required first if we are to exploit that vulnerability to tackle infection. In the case of zinc, we are only just starting to understand how bacteria have evolved to survive in zinc poor environments and studies thus far, have been limited to a small number of bacterial species and model organisms. Here we present the first detailed study of the cellular response of any *Bcc* species to zinc deprivation.

Cellular zinc quotas in *B. cenocepacia* H111 are significantly reduced when grown in a zinc depleted medium, despite no measurable effect of zinc depletion on growth rates. This is surprising, given that 5%–6% of all proteins in any predicted bacterial genome are thought to bind Zn [10], and with approximately 7,500 protein coding genes, the expectation was that *B. cenocepacia* H111 would have a relatively high demand for zinc, given its large genome size for a bacterium. This result suggests that *B. cenocepacia* either maintains significant cellular stores of zinc in replete environments or uses a mechanism of zinc sparing to prioritise limited bioavailable zinc to essential cell functions. The latter is supported by our transcriptomics data, which shows that the expression of several genes encoding usually zinc-dependent proteins are downregulated in response to zinc depletion.

Interestingly, the gene that shows the greatest fold increase in expression when cells are starved of zinc encodes a hypothetical protein, that we have identified as a putative member of the COG0523 subfamily of P-loop GTPases. This is significant as these proteins have been shown to play a role in the response of other bacterial species to zinc limitation, and potentially function as zinc metallochaperones [31, 32]. In *B. cenocepacia* H111, this gene is located in a cluster of genes that were also upregulated in response to zinc-depletion, and that encode proteins that are usually zinc-binding. Based on this finding we hypothesise that this COG0523 protein plays a key role in the response of *B. cenocepacia* H111 to zinc deprivation, and that it functions as a zinc-metallochaperone to deliver zinc to essential proteins.

Also upregulated in response to zinc depletion were a number of genes encoding transport proteins, including an ATP-binding cassette transporter that we predict is the ZnuABC Zn^2+^ importer in this species. The *znuABC* genes are positioned downstream of a *fur-family transcriptional regular*, that is the likely zinc-dependent regulator *zur*, and a regulatory Zur-binding site. It is important to note that it can be difficult to accurately predict the metal specificity of ABC-type transporters from primary sequence data alone, and this is the first experimental evidence that this is the *bona fide* ZnuABC system in *B. cenocepacia* H111. Two genes encoding TonB-dependent outer membrane receptors were also significantly upregulated in response to zinc-depletion, and we hypothesise that one or both plays a role in zinc uptake across the outer cell membrane, although further investigation is required to confirm that or otherwise.

Overall, the data reveals that *B. cenocepacia* responds to zinc depletion in three significant ways, 1) by increasing zinc uptake through the upregulation of genes encoding zinc importers including ZnuABC, 2) by reducing the expression of some non-essential zinc-dependent genes (so called zinc sparing), and 3) through increased expression of a putative zinc metallochaperone of the COG0523 subfamily of P-loop GTPases, presumably to prioritise zinc supply to essential cell functions.

A remaining question concerns effect of zinc bioavailability on the overall virulence and pathogenicity of *Burkholderia* species, and the potential of pharmaceutical based zinc restriction to tackle *Bcc* infections. A proof-of-concept study has demonstrated the potential of the periplasmic zinc binding protein ZnuA as a novel drug target in Gram-negative bacteria [35]. In *S. enterica* serovar Typhimurium, and other Gram-negative pathogens that infect the gastrointestinal tract, ZnuABC is the only high-affinity zinc transporter present. Deletion of *znuA* or *znuABC* in *Salmonella* causes a growth defect in zinc depleted media compared to wild-type strains [35, 36], and the lethal dose of *ΔznuABC* strains is several orders of magnitude higher than wild-type in a murine model [36]. This underscores the potential of zinc uptake systems as novel targets for antimicrobial drugs in Gram-negative bacteria generally. However, in other species of bacteria redundant zinc importers may be present, reducing the effectiveness of targeting a single protein such as ZnuA. A *znuA* mutant strain of *Pseudomonas aeruginosa*, another opportunistic pathogen that like *B. cenocepacia* colonises the lungs of CF patients, grows efficiently in media containing only trace amounts of zinc, which is likely due to the presence additional mechanisms of zinc uptake [37]. This study is therefore important as it provides the first detailed analysis of zinc-homeostasis in a *Bcc* species, which is a prerequisite for the development of any novel antimicrobials targeting zinc homeostasis in this species.

In addition to targeting zinc uptake pathways, metal chelating compounds could also be used to target essential zinc dependent enzymes within the cell. A study investigating the mechanism of action of dithiolopyyrolones, found that the antimicrobial properties of these compounds stem from their ability to chelate intracellular zinc [38], and the use of metal chelators as antimicrobials is an active area of research [39].

## Summary Table

### What Is Known About This Subject

• *B. cenocepacia* is an opportunistic pathogen, responsible for severe respiratory infections in people with cystic fibrosis.
• These bacteria have significant intrinsic antimicrobial resistance, making infections extremely difficult to treat.
• Bacterial zinc uptake and homeostasis systems are attractive targets for the development of new antibiotic drugs.

### What This Paper Adds

• *B. cenocepacia* is remarkably well adapted for growth in zinc poor environments.
• A putative High-affinity ZnuABC zinc uptake system has been identified in this species.
• A putative zinc chaperone (COG0523) has been identified, that is hypothesised to function in zinc trafficking in the cell.

## Summary Sentence

This work represents an advance in biomedical science because it provides the first detailed study of zinc homeostasis in *Burkholderia cenocepacia*.

## Data Availability

The data for this study have been deposited in the European Nucleotide Archive (ENA) at EMBL-EBI under accession number PRJEB62645.
