# Supplementary material for: Transcriptional Response of Burkholderia cenocepacia H111 to Severe Zinc Starvation
Source: Br J Biomed Sci. 2023 Sep 26;80:11597. doi: 10.3389/bjbs.2023.11597 (PMC10563805; doi:10.3389/bjbs.2023.11597)
Supplement: Supplementary file 1 [file Table1.DOCX]

**Table S1. List of primers used for RT-qPCR**

| **Gene locus (gene product)** | **Primer name** | **Sequence 5’ – 3 ‘** |
| --- | --- | --- |
| I35_RS06170 (TonB dependent receptor) | H111 RS06170F | ACTATCTGACGTCCAACCGC |
|  | H111 RS06170R | CTGAACGTGGTCTGGCTGAA |
| I35_RS28540 (COG0523) | H111 RS28540F | ATCTGCGGATCGAGGTTGAC |
|  | H111 RS28540R | ATCGCGCAGGAAATTGAACG |
| I35_RS13365 (Zur) | H111 RS13365F | TCGAACACGGCTTCATCCAC |
|  | H111 RS13365R | CCTGGTGGTGGACTTCGAAA |
| I35_RS13370 (ZnuA) | H111 RS13370F | TGATCTACAACAGCCAGGCC |
|  | H111 RS13370R | TTACTTGCCGGCTGAGAGTG |
| I35_RS13375 (ZnuC) | H111 RS13375F | CCGGACAAGCGGATCATCTT |
|  | H111 RS13375R | CGAATAGGTCCAGTGCACGT |
| I35_RS25975 (Diaminopimelate decarboxylase) | H111 RS25975F | GTATTCACGGCCGACCTGAT |
|  | H111 RS25975R | CGGTGTTGGTCTTGTTGCTG |
| I35_RS16220 (DNA gyrase subunit B) | H111 RS16220F | CGAAAACGTGCTGTGCTTCA |
|  | H111 RS16220R | ACCAGCTTGTCCTTCGTCTG |
